# Stereotactic ablative radiotherapy or best supportive care in patients with localized pancreatic cancer not receiving chemotherapy and surgery (PANCOSAR): a nationwide multicenter randomized controlled trial according to a TwiCs design

**DOI:** 10.1186/s12885-022-10419-4

**Published:** 2022-12-29

**Authors:** D. Doppenberg, M. G. Besselink, C. H. J. van Eijck, M. P. W. Intven, B. Groot Koerkamp, G. Kazemier, H. W. M. van Laarhoven, M. Meijerink, I. Q. Molenaar, J. J. M. E. Nuyttens, R. van Os, H. C. van Santvoort, G. van Tienhoven, H. M. Verkooijen, E. Versteijne, J. W. Wilmink, F. J. Lagerwaard, A. M. E. Bruynzeel

**Affiliations:** 1 Amsterdam UMC, Location Vrije Universiteit Amsterdam, Department of Radiation Oncology, Amsterdam, The Netherlands; 2 Amsterdam UMC, Location University of Amsterdam, Department of Surgery, Amsterdam, The Netherlands; 3 Cancer Center Amsterdam, Amsterdam, The Netherlands; 4 Department of Surgery, Erasmus MC Cancer Institute, Rotterdam, The Netherlands; 5 Department of Radiation Oncology, Regional Academic Cancer Center Utrecht, University of Utrecht, Utrecht, The Netherlands; 6 Amsterdam UMC, Location Vrije Universiteit Amsterdam, Department of Surgery, Amsterdam, The Netherlands; 7 Amsterdam UMC, Location University of Amsterdam, Department of Medical Oncology, Amsterdam, The Netherlands; 8 Amsterdam UMC, Location Vrije Universiteit Amsterdam, Department of Intervention Radiology, Amsterdam, The Netherlands; 9 Department of Surgery, Regional Academic Cancer Center Utrecht, University of Utrecht, Utrecht, The Netherlands; 10 Department of Radiation Oncology, Erasmus MC Cancer Institute, Rotterdam, The Netherlands

**Keywords:** Pancreatic cancer, Radiotherapy, SABR, SBRT, MRgRT, Quality-of-life

## Abstract

**Background:**

Significant comorbidities, advanced age, and a poor performance status prevent surgery and systemic treatment for many patients with localized (non-metastatic) pancreatic ductal adenocarcinoma (PDAC). These patients are currently treated with ‘best supportive care’. Therefore, it is desirable to find a treatment option which could improve both disease control and quality of life in these patients. A brief course of high-dose high-precision radiotherapy i.e. stereotactic ablative body radiotherapy (SABR) may be feasible.

**Methods:**

A nationwide multicenter trial performed within a previously established large prospective cohort (the Dutch Pancreatic cancer project; PACAP) according to the ‘Trial within cohorts’ (TwiCs) design. Patients enrolled in the PACAP cohort routinely provide informed consent to answer quality of life questionnaires and to be randomized according to the TwiCs design when eligible for a study. Patients with localized PDAC who are unfit for chemotherapy and surgery or those who refrain from these treatments are eligible. Patients will be randomized between SABR (5 fractions of 8 Gy) with ‘best supportive care’ and ‘best supportive care’ only. The primary endpoint is overall survival from randomization. Secondary endpoints include preservation of quality of life (EORTC-QLQ-C30 and -PAN26), NRS pain score response and WHO performance scores at baseline, and, 3, 6 and 12 months. Acute and late toxicity will be scored using CTCAE criteria version 5.0: assessed at baseline, day of last fraction, at 3 and 6 weeks, and 3, 6 and 12 months following SABR.

**Discussion:**

The PANCOSAR trial studies the added value of SBRT as compared to ‘best supportive care’ in patients with localized PDAC who are medically unfit to receive chemotherapy and surgery, or refrain from these treatments. This study will assess whether SABR, in comparison to best supportive care, can relieve or delay tumor-related symptoms, enhance quality of life, and extend survival in these patients.

**Trial registration:**

Clinical trials, NCT05265663, Registered March 3 2022, Retrospectively registered.

## Background

For pancreatic ductal adenocarcinoma (PDAC), surgery in combination with (neo) adjuvant systemic therapy as curative treatment, is feasible in only 15–20% of patients [1–3]. In a cohort of 3090 patients newly diagnosed with PDAC in the Netherlands in 2014–2015, almost 60% of patients did not receive any antitumor treatment, irrespective of stage [4]. A disturbing finding is that 30% of patients who did not undergo treatment, had localized (i.e. non-metastatic) clinical stage I-III disease. Among these patients, a higher median age and a higher incidence of relevant co-morbidity was observed. The main reasons for withholding treatment were patient’s choice (27%), extensive disease (21%) and functional status (15%). Among the patients for whom high age (mean age 86 years) was mentioned as the main reason to withhold treatment, 37% had a clinical stage I tumor. The median survival of these frail patients with localized PDAC was found to be only 1–4 months (with variable reasons to withhold from treatment). This is comparable to other studies reporting a median overall survival of 2–4 months in patients with localized and metastatic disease treated with best supportive care only [3, 5].

Considering this, there is a substantial group of patients with localized PDAC either medically unfit to undergo surgery and chemotherapy, or both. Additionally, there is a group whom refrain from chemotherapy and surgery because of expected toxicity and adverse events which reasonably occur in higher rates among patients with higher age [6]. These two groups of patients may be sufficiently fit to undergo a short course of stereotactic ablative radiotherapy (SABR). SABR is a non- invasive radiation treatment that is delivered with high precision to the primary tumor in only a few fractions, resulting in a high biological dose to the primary tumor, relatively sparing surrounding organs at risks (OARs) [7–10].

It is hypothesized that in comparison to the current standard of care, which exists of best supportive care, SABR may relieve or delay tumor-related symptoms, preserve or improve quality of life, and extend survival in elderly and frail patients with localized PDAC. A systematic review of SABR in a heterogeneous population of patients with LAPC (with or without chemotherapy) showed a one year local control rate after SABR of 72.3% (95% confidence interval 58.5%–79%) [11]. Furthermore, acute toxicity grade > 3 ranged from 0 to 36% with only 3 out of 19 studies showed grade ≥ 3 acute toxicity of more than 10%.

Previous smaller studies showed the potential of SABR to safely treat patients with PDAC who could not tolerate surgery, chemotherapy or several weeks of chemoradiotherapy. Overall median OS ranged between 6.4–8 months, with grade ≥ 3 adverse events ranging from 0 to 15% in these elderly and medically inoperable patients [12–14]. These studies reported high rates of symptom relief (70–80%) following SABR, especially in patients who experienced nausea and abdominal pain before the start of the SABR. Considering this, SABR has the potential to decrease symptoms using short overall treatment times, and therefore may be an attractive option for patients who otherwise would only receive best supportive care. However, evidence that SABR is superior to best supportive care in these patients is lacking, by the conduction of this study we aim to evaluate this.

## Methods/design

The PANCOSAR study, in which patients with pancreatic cancer, with initially localized disease who are medically unfit for chemotherapy and surgery or choose to refrain from these treatments, are randomized between 5 × 8 Gray (Gy) SABR or best supportive care, is conducted as a nationwide, multicenter randomized trial according to the ‘Trials within Cohorts design’, hereafter TwiCs design [15, 16]. PANCOSAR is performed within the Dutch Pancreatic Cancer Project (PACAP) which is a prospective observational cohort in which all 48 centers of the Dutch Pancreatic Cancer Group (DPCG) participate [17, 18]. All patients with PDAC are eligible for inclusion in this PACAP cohort, a national registration outcome project that also provides the Dutch Pancreatic Cancer Audit (DPCA), a nationwide Expert Panel and the Dutch Pancreas Biobank (PancreasParel) [19, 20]. Participants in the PACAP-cohort have provided informed consent for the purpose of collecting data on demographics, quality of life, and clinicalfindings during follow-up. Additionally, patients have provided informed consent for potential current or future randomization in (TwiCs design) clinical trials if they are or become eligible. In such a trial, once a patient is randomized, additional informed consent will be asked for any intervention that is not considered standard treatment, so called staged informed consent [21]. The TwiCs design aims to improve the inclusion of patients, to limit selection and crossover bias and to prevent potential distress in patients being randomized for the control group. In addition, this design allows optimal use of data that are already obtained through the PACAP cohort. The implementation of the PANCOSAR study within PACAP according to the TwiCs design, was approved upon ethical assessment, reference number NL72181.029.21.

**Fig. 1:**
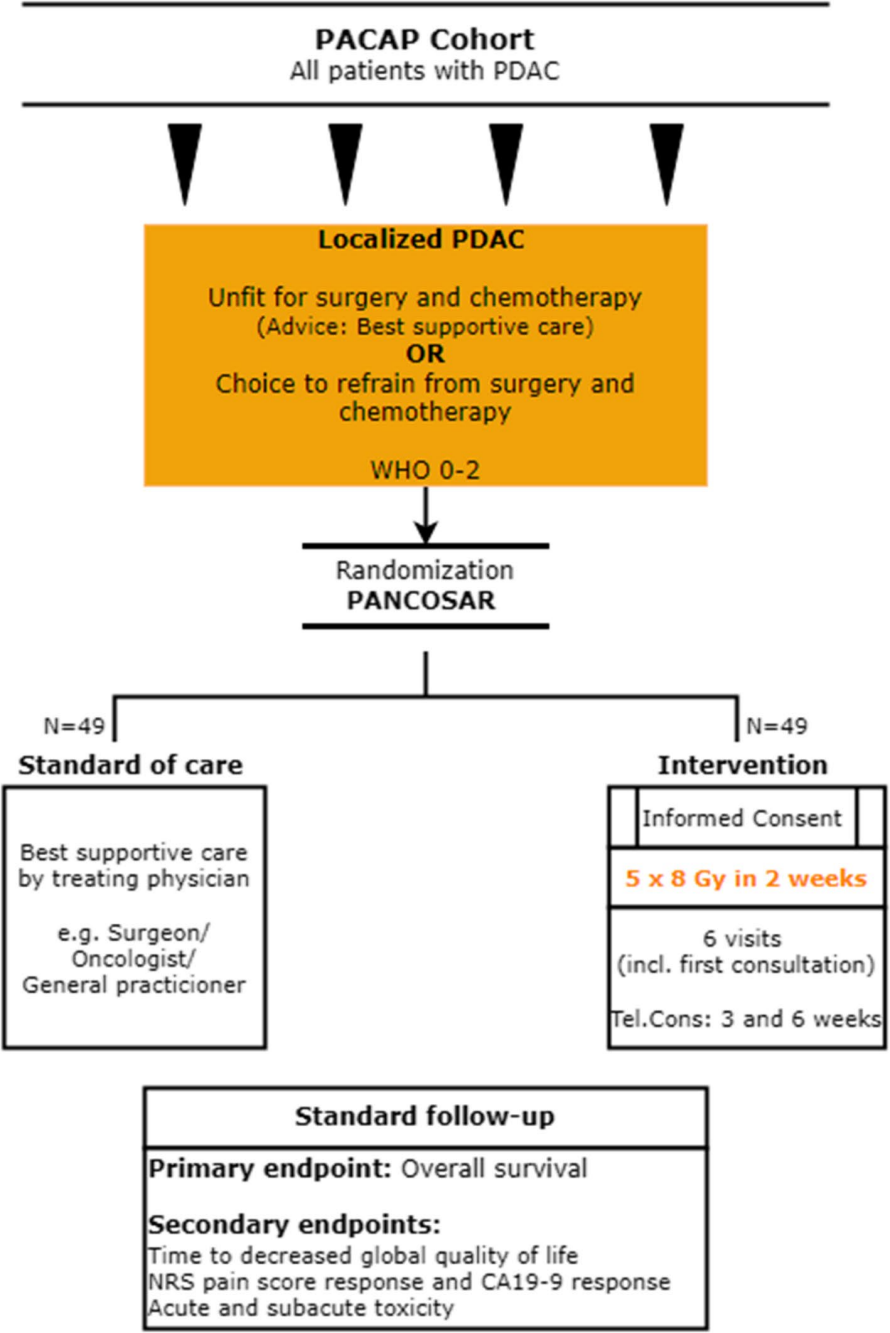
Flow diagram PANCOSAR study

To investigate whether SABR, in comparison to best supportive care, relieves tumor-related symptoms, enhances quality of life, and prolongs survival, patients will be randomized between either SABR with ‘best supportive care’ and ‘best supportive care’ only by the principal investigator or coordinating physician. Following the TwiCs design, patients allocated to the intervention arm receive an appointment with the radiation oncologist to confirm eligibility and subsequently are asked additional informed consent. Patients randomized in the control arm will not be approached since they gave informed consent for the PACAP cohort (see Fig. 1).

All PACAP-participants with pathologically confirmed primary localized PDAC unfit for or refraining from surgery or chemotherapy, or both, with a WHO performance score ≤ 2 are eligible for inclusion. Patients must be able to provide written informed consent.

In case treatment with chemotherapy is stopped, i.e. due to intolerability, a patient may be included in the study if no more than two cycles (i.e. two months or treatment) were administered.

Pathologically confirmation of the PDAC should be obtained. However, in case multiple attempts to obtain pathological confirmation fail, consensus on presence of local PDAC and trial eligibility can be obtained in a multidisciplinary meeting (e.g.; based on clinical situation, imaging, and elevated CA 19–9).

Exclusion criteria for participation are distant metastasis, imminent bowel obstruction, active bleeding and uncontrolled infection. In the case of MR-guided radiotherapy certain MRI related contra-indications exist such as presence of a pacemaker, implantable cardioverter defibrillator or severe claustrophobia.

**Fig. 2:**
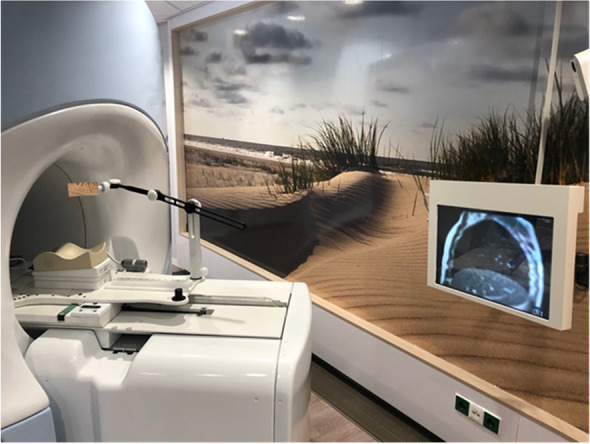
The visual feedback system, including an MR-compatible monitor at the head end of the MR bore and an adjustable mirror (Botman et al., Clin Transl Radiat Oncol. 2019)

**Fig. 3:**
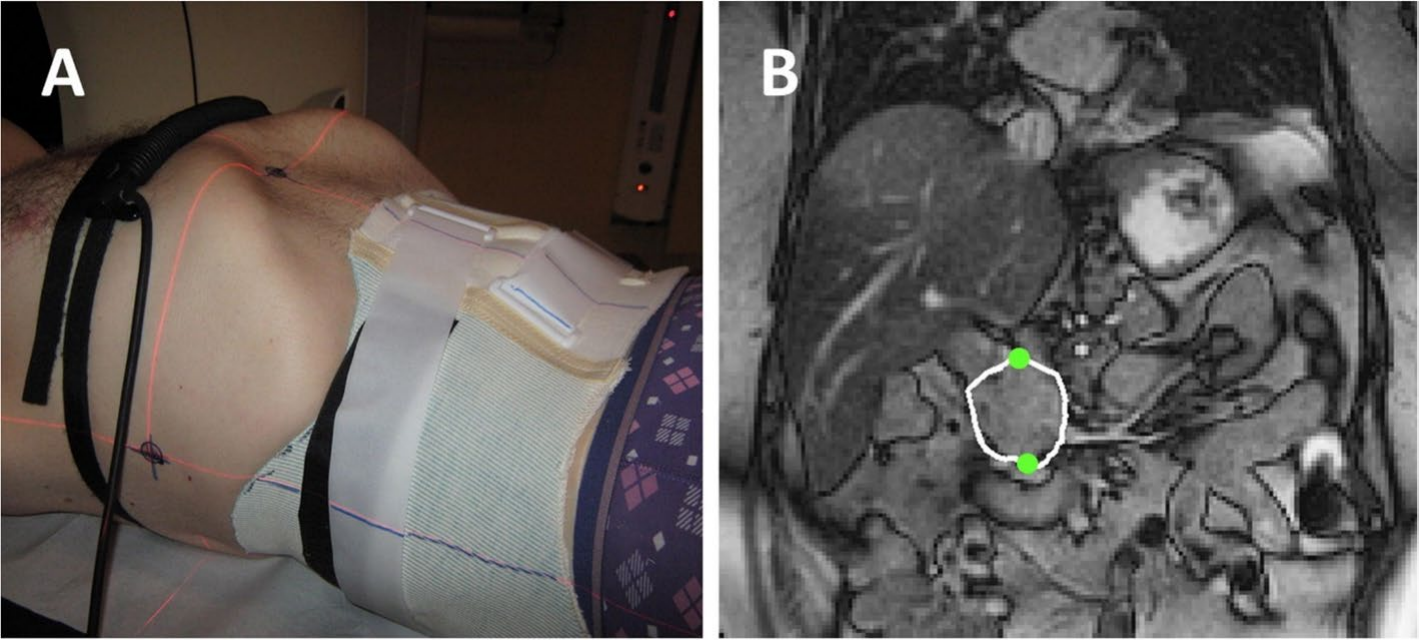
Custom-made abdominal corset (Heerkens et al. Physics and imaging in Radiation oncology. 2017)

Using an image-guided hypo fractionated scheme SABR will be delivered in 5 fractions of 8 Gy (total dose 40 Gy), prescribed to 95% of the planning target volume (PTV). Within a maximum treatment period of 14 days, radiation is delivered on alternate days. Treatment will be delivered in centers with extensive experience in delivering SABR for PDAC. In case of CT-guided delivery, the insertion of fiducial markers in the pancreatic tumor is required, prior to the planning CT scan; for MR-guided delivery this is not necessary. All patients will have a planning CT-scan in preparation for treatment delivery, and in case of MR guided treatment a planning-MRI is performed in treatment position. Patients will be simulated and treated in supine position according to institutional standards. For delivery of SABR including a visual feedback system, an MR-compatible monitor at the head end of the MR bore and an adjustable mirror is used (Fig. 2). For delivery in reduced breathing motion a custom-made abdominal corset is used (Fig. 3).

For this study, the clinical target volume (CTV) equals the gross tumor volume (GTV), including the tumor in the pancreas and immediately adjacent involved lymph nodes. The planning target volume (PTV) will be generated by adding a margin of 3–5 mm around the GTV depending on institutional protocols. The duodenum, bowel stomach, liver, kidneys, and spinal cord will be contoured as avoidance structures. Limits of the maximum doses to critical structures such duodenum, bowel and stomach are prioritized.

Generally, patients receive dietary instructions as treatment will be delivered after 2 hours fasting. Patients may be pre-medicated (2 hours before each fraction) with ondansetron and/or dexamethasone in order to prevent radiation-induced early side effects, or aggravation of pain. In case of intolerance or severe complaints, modification or discontinuing the radiation plan can be necessary.

All radiation-associated early and late toxicity will be scored according to the NCI-CTCAE toxicity criteria version 5.0 [22]. Potential acute toxicity (within three months) that particularly will be noted are fatigue, pain, nausea, vomiting and diarrhea. Regarding toxicity after three months (late toxicity), particular attention will be paid to complaints of the stomach or duodenum, complaints, such as dyspepsia, bleeding, or perforation.

After completion of the last SABR fraction, questionnaires will be filled in, and all end-of-treatment forms. Patients must be seen by the radiation oncologist at the day of the last fraction. Patients in the SABR arm will be contacted additionally at 3 and 6 weeks by the radiation oncologist for acute toxicity. Follow-up with respect to disease status, performance status and quality of life will be performed by telephone and (e-)mail through PACAP at the fixed study assessment points, which coincide with the time points for follow up during best supportive care.

Primary endpoint is the overall survival rate from the date of randomization. Secondary endpoints include time to a decrease in global quality of life (QoL, using the QLQ- C30 and EORTC-PAN26 questionnaires), pain response by using the Numerical rating Scale (NRS), CA19.9 response, and acute and subacute toxicity rates using common toxicity criteria (NCI-CTCAE toxicity criteria version 5.0).

Based on previous literature and our own experience, we assume that at 6 months from the date of randomization, 50% of patients treated with SABR will be alive versus 20% of patients managed with best supportive care [4, 11]. Taking into account that an estimated 30% of patients randomized for the intervention arm will ultimately refrain from SABR, this will dilute the expected survival in the intervention arm to 41% (0.7 × 50% + 0.3 × 20%). Since the control arm is not informed about the intervention, we assume 100% compliance in this arm. Because this phase 2 study will constitute preliminary outcomes for further studies, it is considered not as bad to unjustly reject the null hypothesis (i.e. Finding an effect that actually exists is more important than to wrongly find an effect). Therefore we use and alpha of 15% instead of 5%. Following calculation of the sample size with alpha 15% and power 80%, 49 patients per treatment arm are required in this randomized trial [23].

Randomization will be stratified according to the reason for not undergoing surgery and/or systemic chemotherapy in order to ensure an even distribution of patients with potentially better prognosis who had refused chemotherapy and/or surgery in both study arms. Patients will be randomized using a secured online computer controlled permuted-block randomization in a 1:1 ratio (CASTOR EDC, CIWIT B.V., Amsterdam, The Netherlands). The block sizes itself will are prone to randomly change, varying between 4, 6 and 8 patients.

According to the TwiCs design, patients will be informed about randomization to the intervention (SABR) within this trial by either the local principal investigator or the coordinating physician. Furthermore, they will be informed that they are free to decide whether they want to adhere or deny this intervention. If they choose to adhere to the assigned treatment arm, written informed consent will be obtained in addition to verbal information, by the local principal investigator before the start of SABR. After signing informed consent, patients should receive the first fraction of SABR within four weeks. Participants in the PACAP-cohort who have been randomized for the control arm, i.e. best supportive care, will be treated and followed according to the current standard of care.

Data will be collected using a secured electronic database (CASTOR EDC, CIWIT B.V., Amsterdam, The Netherlands) by the coordination physician and a clinical data manager. All variables mentioned in the inclusion criteria will be analyzed and reported using standard descriptive statistics. Continuous variables will be summarized with standard statistics including means, standard deviations, medians and ranges. Categorical variables will be summarized with frequencies. When appropriate, box plots and cross tables will be used for descriptive statistics of continuous and categorical variables, respectively. Survival endpoints will be analyzed using log rank tests and Kaplan Meier plots, additionally Cox proportional hazards models are used in order to adjust for stratification and prognostic variables. *P*-values below 0.05 will be considered significant. All calculations will be generated by statistical package for social sciences software (IBM SPSS v.28).

Data will be handled confidentially. An individual subject identification code is used to link the data to the subject. A code is generated based on the first three letters of the month of inclusion and a sequential number. The study coordinators safeguard the key to the code and access to the coded data will be restricted to the principal investigators, the coordinating investigator, the study coordinators and the monitor. The handling of personal data will comply with the General Data Protection Regulation (in Dutch: Algemene vordering gegevensbescherming (AVG)). The principal investigators will keep the source data for 15 years.

Any modifications to the protocol which may impact on the conduct of the study, patients safety, potential benefit of the patient, including changes of study objectives, inclusion criteria, sample sizes, study procedures, will require an amendment to the protocol. If the study staff will concur with such amendment, approval of the Institutional Medical Ethics Review board of VU University Medical Center in Amsterdam is necessary prior to implementation. Important protocol modification will be communicated by electronic newsletters and additional site visits if necessary.

To preserve safety and assess the hypothesis that SABR is safe and feasible in the intended population, an interim analysis will be conducted after 36 patients have completed the follow-up assessments after three months. This includes an epidemiological assessment of all adverse events (AE’s) and primary and secondary outcome measures. Using prior literature as reference, we have decided that the study will be halted in case of an incidence of grade ≥ 3 GI toxicity of > 20% in the intervention arm [11]. All grade > 2 AEs will be reported up to three months following SBRT, only the treatment induced grade > 2 AEs will be recorded up to the end of the study. The principal investigator or an authorized delegate will decide whether or not an AE is related to the SABR. If an AE has occurred, exact information on the time period, magnitude of the event and consequences for the patients will be investigated.

An independent data safety and monitoring committee (DSMB) has been assigned to examine safety parameters and evaluate the progress of the study. The DSMB consists of an independent epidemiologist/statistician (chairman), a radiation oncologist and a surgeon. All involved physicians will be repeatedly urged to report any potential AE’s. The monitoring committee will review and debate this list of AEs. To discuss a specific AE, the monitoring committee may call for a comprehensive report. A copy of this report will be sent to the involved physicians and the main ethics board.

Periodic monitoring visits will be planned by an independent monitoring committee The Clinical Research Bureau (CRB) of VUmc will monitor the safety of the trial and the storage of the data. Monitoring visits will be scheduled throughout the course of the study, and at frequencies thought suitable, at mutually convenient times, stated in the monitor plan.. These visits will are made to assess the progress of the study, to guarantee the subjects’ rights and wellbeing, to confirm that the reported clinical study data are accurate, complete and verifiable from source documents, and to determine whether the study’s conduct is in accordance with the protocol and any approved amendments, good clinical practice, and applicable national regulatory requirements.. During a monitoring visit the investigator and staff will review the crucial clinical study documentation. During these visits, the investigator and staff should be accessible to assist the evaluation of the clinical study records and to discuss, address, and document any discovered discrepancies.

## Discussion

Based on prior literature, a short course of high-dose precise radiotherapy, SABR, is likely to be feasible even in elderly and/or frail patients with PDAC for whom the current standard of treatment is best supportive care [11, 13, 24]. The potential benefit of this strategy for patients is that it has a reasonable chance of a durable local control of the disease, could relieve symptoms such as pain, and may potentially prolong survival. Since the use of small uncertainty margins with consequent limitation of high radiation dose to OARs, it is anticipated that toxicity will be low and therefore acceptable for this patient group.

Although prior studies are promising and support a PANCOSAR-like trial, the conduct is not without concerns. To start with, the study design according to TwiCs is a novel design within the DPCG and it is unknown whether this design succeeds for the destined frail patient group and patients refraining from surgery and/or chemotherapy. However, the destined study population is in need for new treatment options as no treatment other than best supportive care is available. Considering this, a quick conduction and completion of a trial is desirable. The TwiCs design has multiple features that may allow us to do so. First, the PANCOSAR study is performed within the PACAP cohort, consisting currently of patients from 48 participating centers, which enhances rapid implementation of the study within the existing infrastructure of this cohort. Second, a TwiCs design has the potential to enroll higher proportions of eligible patients and subsequently may increase generalizability [25]. It is known that conventional randomized controlled trials can be costly and time consuming as they suffer from slow and inefficient accrual due to patient related reasons [26]. Main motives for patients to refrain from conventional RCTs are aversion for randomization, difficulties understanding the concept of RCT and preference for allocation to a specific study arm [26]. Declining participation due to preferences of treatment allocation may introduce bias and limit external validity as a proportion of eligible patients will not be included [26, 27]. Lastly, the TwiCs design removes the burden for patients to be allocated to the non-preferred arm, which is an important reason to conduct the PANCOSAR study within this design. Clinicians may argue it can be ethically difficult to withhold patients from information about existing trials when patients ask for study treatment options. To encounter this problem, it is important to broadly inform health care professionals about the TwiCs design and appropriately apply the staged-informed consent procedure at time of enrollment of the cohort [21].

Another concern for this trial is the intention to conduct this in a patient population with pathologically confirmed PDAC. Obtaining pathology prior to randomization may be troublesome and even unsafe for some patients, particularly in our destined frail group. With the knowledge that patients can be allocated to the control arm, it can be an ethical dilemma for doctors to obtain pathological proof. Therefore, we have decided that in the case it is considered unsafe for a patient or obtaining biopsies failed, consensus for randomization can be obtained on presence of local PDAC and trial eligibility discussed in a multidisciplinary meeting.

Based on extensive literature regarding SABR for PDAC and the use of relatively small uncertainty margins with consequent limitation of radiation dose to OARs, we expect that toxicity will be acceptable in the intervention arm. Additional safety measures for the intervention arm include a planned interim analysis and installation of a DSMB. Following this, it is hypothesized that treatment with high-dose precise radiotherapy may postpone a decrease in global QoL, which an essential secondary endpoint of the study. Since PACAP facilitates valid questionnaires assessing QoL in the entire cohort, this study design allows adequate outcome comparison of both arms without additional study specific procedures for the control arm. For the control arm no anticipated disadvantages are expected since management in the control arm with best supportive care is equal to current standard.

Regarding prolongation of life, preservation of QoL and the desire for a non- or minimally invasive treatment for patients with localized PDAC with no other treatment options than best supportive care, SABR has the potential to be introduced as a safe and feasible treatment option in the future. However, valid evidence concerning this hypothesis is lacking and the conduct of this study is necessary to appropriately assess this, respecting the frail study population.

## Data Availability

Data sharing is not applicable to this article as no datasets were generated or analyzed during the current study.

## Abbreviations

PDAC: Pancreatic ductal adenocarcinoma
LAP: Locally advanced pancreatic cancer
SABR: Stereotactic ablative body radiotherapy
PACAP: Dutch pancreatic cancer project
DPCG: Dutch pancreatic cancer group
DPCA: Dutch pancreatic cancer audit
Pancreasparel: Dutch pancreas biobank
TwiCs: Trial within cohorts
Gy: Gray
PTV: Planning target volume
CTV: Clinical target volume
GTV: Gross tumor volume
OAR: Organs at risk
QoL: Quality of life
NRS: Numeric rating scale
AE’s: Adverse events
DSMB: Data safety monitoring board
WMO: Wet medisch-wetenschappelijk onderzoek met mensen
