# Correction: Stereotactic ablative radiotherapy or best supportive care in patients with localized pancreatic cancer not receiving chemotherapy and surgery (PANCOSAR): a nationwide multicenter randomized controlled trial according to a TwiCs design

**DOI:** 10.1186/s12885-023-10521-1

**Published:** 2023-01-20

**Authors:** D. Doppenberg, M. G. Besselink, C. H. J. van Eijck, M. P. W. Intven, B. Groot Koerkamp, G. Kazemier, H. W. M. van Laarhoven, M. Meijerink, I. Q. Molenaar, J. J. M. E. Nuyttens, R. van Os, H. C. van Santvoort, G. van Tienhoven, H. M. Verkooijen, E. Versteijne, J. W. Wilmink, F. J. Lagerwaard, A. M. E. Bruynzeel, for the Dutch Pancreatic Cancer Group

**Affiliations:** 1 Amsterdam UMC, Location Vrije Universiteit Amsterdam, Department of Radiation Oncology, Amsterdam, The Netherlands; 2 Amsterdam UMC, Location University of Amsterdam, Department of Surgery, Amsterdam, The Netherlands; 3 Cancer Center Amsterdam, Amsterdam, The Netherlands; 4 Department of Surgery, Erasmus MC Cancer Institute, Rotterdam, The Netherlands; 5 Department of Radiation Oncology, Regional Academic Cancer Center Utrecht, University of Utrecht, Utrecht, The Netherlands; 6 Amsterdam UMC, Location Vrije Universiteit Amsterdam, Department of Surgery, Amsterdam, The Netherlands; 7 Amsterdam UMC, Location University of Amsterdam, Department of Medical Oncology, Amsterdam, The Netherlands; 8 Amsterdam UMC, Location Vrije Universiteit Amsterdam, Department of Intervention Radiology, Amsterdam, The Netherlands; 9 Department of Surgery, Regional Academic Cancer Center Utrecht, University of Utrecht, Utrecht, The Netherlands; 10 Department of Radiation Oncology, Erasmus MC Cancer Institute, Rotterdam, The Netherlands

**Correction: BMC Cancer 22, 1363 (2022)**

**https://doi.org/10.1186/s12885-022-10419-4**

Following publication of the original article [1], the authors reported an error in the author group. The text “for the Dutch Pancreatic Cancer Group” was missing from the list of authors. This has been updated in the author group above and the original article [1] has been updated.
